# Acrolein adducts and responding autoantibodies correlate with metabolic disturbance in Alzheimer’s disease

**DOI:** 10.1186/s13195-023-01261-2

**Published:** 2023-06-22

**Authors:** Monika Renuka Sanotra, Shu-Huei Kao, Ching-Kuo Lee, Chun-Hsien Hsu, Wen-Chung Huang, Tsuei-Chuan Chang, Fang-Yu Tu, I-Uen Hsu, Yung-Feng Lin

**Affiliations:** 1 Ph.D. Program in Medical Biotechnology, College of Medical Science and Technology, Taipei Medical University, Taipei, 110 Taiwan; 2 School of Medical Laboratory Science and Biotechnology, College of Medical Science and Technology, Taipei Medical University, Taipei, 110 Taiwan; 3 School of Pharmacy, College of Pharmacy, Taipei Medical University, Taipei, 110 Taiwan; 4 Department of Family Medicine, Taipei City Hospital, Heping Fuyou Branch, Taipei, 100 Taiwan; 5 Department of Family Medicine, Cardinal Tien Hospital, New Taipei, 231 Taiwan; 6 School of Medicine, College of Medicine, Fu Jen Catholic University, New Taipei, 242 Taiwan; 7 Graduate Institute of Health Industry Technology, College of Human Ecology, Chang Gung University of Science and Technology, Taoyuan, 333 Taiwan; 8 Department of Neuroscience, Yale University School of Medicine, New Haven, CT 06510 USA

**Keywords:** Alzheimer’s disease, Amyloid-beta, Acrolein adduct, Metabolic syndrome, Autoantibody

## Abstract

**Background:**

Alzheimer’s disease (AD) is caused by many intertwining pathologies involving metabolic aberrations. Patients with metabolic syndrome (MetS) generally show hyperglycemia and dyslipidemia, which can lead to the formation of aldehydic adducts such as acrolein on peptides in the brain and blood. However, the pathogenesis from MetS to AD remains elusive.

**Methods:**

An AD cell model expressing Swedish and Indiana amyloid precursor protein (APP-Swe/Ind) in neuro-2a cells and a 3xTg-AD mouse model were used. Human serum samples (142 control and 117 AD) and related clinical data were collected. Due to the involvement of MetS in AD, human samples were grouped into healthy control (HC), MetS-like, AD with normal metabolism (AD-N), and AD with metabolic disturbance (AD-M). APP, amyloid-beta (Aß), and acrolein adducts in the samples were analyzed using immunofluorescent microscopy, histochemistry, immunoprecipitation, immunoblotting, and/or ELISA. Synthetic Aß_1-16_ and Aß_17-28_ peptides were modified with acrolein in vitro and verified using LC–MS/MS. Native and acrolein-modified Aß peptides were used to measure the levels of specific autoantibodies IgG and IgM in the serum. The correlations and diagnostic power of potential biomarkers were evaluated.

**Results:**

An increased level of acrolein adducts was detected in the AD model cells. Furthermore, acrolein adducts were observed on APP C-terminal fragments (APP-CTFs) containing Aß in 3xTg-AD mouse serum, brain lysates, and human serum. The level of acrolein adducts was correlated positively with fasting glucose and triglycerides and negatively with high-density lipoprotein-cholesterol, which correspond with MetS conditions. Among the four groups of human samples, the level of acrolein adducts was largely increased only in AD-M compared to all other groups. Notably, anti-acrolein-Aß autoantibodies, especially IgM, were largely reduced in AD-M compared to the MetS group, suggesting that the specific antibodies against acrolein adducts may be depleted during pathogenesis from MetS to AD.

**Conclusions:**

Metabolic disturbance may induce acrolein adduction, however, neutralized by responding autoantibodies. AD may be developed from MetS when these autoantibodies are depleted. Acrolein adducts and the responding autoantibodies may be potential biomarkers for not only diagnosis but also immunotherapy of AD, especially in complication with MetS.

**Supplementary Information:**

The online version contains supplementary material available at 10.1186/s13195-023-01261-2.

## Introduction

Alzheimer’s disease (AD) is the most widespread dementia and afflicts aging populations worldwide. The cause of AD is the result of many intertwining pathologies. However, progressive neuronal loss found in AD is theorized to result from the accumulation of extracellular amyloid plaques comprised of amyloid-ß (Aß) which is generated through proteolytic cleavage of amyloid precursor protein (APP) by ß- and γ-secretases [1]. More and more investigations suggest that AD is strongly correlated with metabolic syndrome (MetS) involving oxidative stress and post-translational modification (PTM) [2–4]. MetS is a group of five conditions, namely obesity, hypertension, hyperglycemia, hypertriglyceridemia, and reduced high-density lipoprotein-cholesterol (HDL-C) in the blood. Imbalanced blood levels of glucose and lipids enhance lipid peroxidation, producing acrolein, 4-hydroxynonenal (HNE), and other aldehydic adducts in AD brain, cerebrospinal fluid (CSF), and blood. Nationwide surveys from Taiwan and Korea also supported that metabolic aberration could be a good indicator to diagnose the development of dementia specifically AD [5, 6]. However, the detailed mechanism of pathogenesis from MetS to AD remains elusive.

Studies suggest that increased lipid peroxidation in membranes causes increased levels of acrolein and HNE in the amygdala, hippocampus/parahippocampal gyrus, and inferior parietal lobule [7]. In fact, acrolein is more neurotoxic than HNE as seen in the primary culture of rat hippocampal neurons. Unfavorable effects of acrolein comprise reactive oxygen species (ROS) formation, protein adduction, inflammation, mitochondrial dysfunction, membrane disruption, endoplasmic reticulum stress, and DNA damage [8]. Thus, acrolein is associated with various diseased conditions and influences the progression of neurodegenerative diseases such as AD. Recently, an acrolein-induced sporadic AD animal model is developed. These mice with chronic exposure to acrolein resemble most of the AD pathologies including high levels of Aß and tau, resulting in cognitive decline and psychological impairments [9, 10]. In only one month, these sporadic AD model mice show neurodegeneration, such as reduced density of dendritic spines in the hippocampal neurons with decreased postsynaptic density 95 and Synapsin1 and activated ROCK2/RhoA/p-cofilin-associate pathway, as well as the proliferation of astrocytes and microglia [9]. The high reactivity of acrolein is due to the presence of carbonyl group (C = O) and double bond (C = C) to react with nucleophiles including the sulfhydryl group of cysteine, imidazole of histidine and ε-amino group of lysine [11]. These amenable amino acid residues are involved in major cellular processes with physiological importance including cellular buffering, redox signaling, ROS sensing, and enzyme catalysis. Therefore, acrolein adduction may lead to substantial alterations in protein function. Also, acrolein can inhibit enzymes that metabolize acrolein-GSH conjugates and xenobiotics, affecting the metabolism of many drugs and pollutants.

Immune responses play a major role in neurodegenerative diseases including AD [12]. Natural antibodies, for example, IgG and IgM levels are linked to related disease progression. Depletion of responding autoantibodies is inversely proportional to the progress and onset of the disease, while an increased level helps in protection [13]. Autoantibodies and their corresponding antigens can be further studied to assess their potential as AD diagnostic biomarkers and therapeutic targets [14]. Indeed, autoantibodies can not only distinguish individuals with AD from non-demented controls but also predict the risk of progression from mild to severe AD [15]. In certain research, anti-Aß IgM level is shown to decrease significantly in the AD group compared to control [16]. In addition, the response of autoantibodies to Aß is suggested to be epitope dependent [17]. Interestingly, in our recent study, both Aß-specific IgG and IgM levels are found to be increased in hyperglycemia as well as in AD, suggesting that Aß peptides may be a general target and responds varyingly in metabolic disorder [18]. Furthermore, dysmetabolic adducts including HNE and Nε-(Carboxyethyl)lysine, an advanced glycation end product, are both increased in AD patients’ blood, and their responding IgM levels are decreased with the disease progression [18, 19]. Therefore, specific metabolites or adducts and their responding IgM may be used as biomarkers for AD diagnosis and treatment.

The main goal of this research is to search for biomarkers and supporting evidence of AD development from metabolic syndrome. Aberrant metabolism is directly linked with Aß, acrolein adducts and responding antibodies which are easily accessible in biological fluids. The methodology developed in this research may be used to determine AD pathology and its severity.

## Materials and methods

### AD cell model

A cellular AD model was established in mouse neuroblastomaneuro-2a cells (ATCC-CCL-131) previously [20, 21]. Among the major isoforms of APP, APP-695 is most abundant in the brain. Neuro-2a cells were transfected with pCAX vector, pCAX-APP-695 (human wild-type APP-695 isoform) or p-CAX-APP-Swe/Ind (human APP-695 K595N/M596L/V642F) (Addgene, Watertown, MA, USA). The cells were cultured and maintained in minimum essential medium (Eagle) with 2 mM L-glutamine, 0.1 mM non-essential amino acids, 2.2 g/L sodium bicarbonate, and Earle’s salt, and supplemented with 10% heat-inactivated fetal bovine serum, 100 units/mL penicillin, 100 µg/mL streptomycin, and 2.5 µg/mL amphotericin B (Thermo Fisher Scientific Inc, Waltham, MA USA). Cell culture plates were incubated at 37 °C with 5% CO_2_. Immunofluorescent microscopy was used to observe cell differentiation and neurite outgrowth. To determine the AD pathology in the cells, sodium dodecyl sulfate–polyacrylamide gel electrophoresis (SDS-PAGE) was performed. One set of the gels was used for immunoblotting to check the levels of APP full-length (APP-FL), APP C-terminal fragments (APP-CTFs), and acrolein adducts; the other set was stained with Coomassie brilliant blue (CBB) (Bio-Rad Laboratories, Hercules, CA, USA) as a loading control.

### AD mouse model

Wild-type (C57BL/6 or B6) and 3xTg-AD mice [B6; 129-Psen1^tm1Mpm^ Tg(APP-Swe, TauP301L)1Lfa/Mmjax] expressing three mutant genes, namely Psen1-M146V, APP-K670N/M671L, and Tau-P301L, were obtained from the Jackson Laboratory (Bar Harbor, ME, USA)and used in previous studies [21]. The AD mice show neurodegenerative signs at the age of 6 months and older. In this study, mice at 2 and 9 months old were used. Mouse memory was tested in Morris water maze. Aß plaques in mouse brain were analyzed using histochemistry. Mouse serum and brain lysates were examined using SDS-PAGE with immunoblotting. All animal procedures in this study were approved by the Institutional Animal Care and Use Committee in Taipei Medical University (LAC-2019–0448).

### Morris water maze

The mice at 2 and 9 months old were trained to find the hidden platform in the target quadrant of the water maze for 4 days. During the testing phase on day 5, the platform was removed, and the time for the mice to spend in the target quadrant in total 2 min was recorded. After tested in the water maze, the mice were sacrificed and dissected to collect the brain and blood tissues.

### Histochemistry

One half of the mouse brain was used for histochemistry and the other half for immunoprecipitation and immunoblotting. The left brain was fixed in 10% formaldehyde at room temperature for 24–36 h and sectioned into 6 µm. After rehydrated with distilled water for 30 s, the brain sections were incubated with alkaline solution for 20 min and then in Congo red for 30 min to display Aß deposition (CIS-Biotechnology Co. Ltd., Taichung, Taiwan). The plaque burden at different ages was quantified using Image J (Wayne Rasband, NIH, USA).

### Human subjects

Human serum samples were collected from Taipei Medical University Hospital and the Joint Biobank in this study. AD samples were from patients diagnosed based on the Diagnostic and Statistical Manual of Mental Disorders (DSM IV), as well as in accordance with criteria from both the National Institute of Neurological and Communicative Disorders (USA) and the Stroke-Alzheimer’s Disease and Related Disorder Association [22, 23]. A subject with mini-mental state examination (MMSE) score below 26 out of 30 was regarded as probable AD [24]. These subjects had no focal neurological abnormalities such as cranial nerve palsy, hemiparesis, incoordination, and gait disturbances. Their brain magnetic resonance imaging and laboratory tests including venereal infection, thyroid function, vitamin B12, and folic acid levels were performed to exclude other causes of dementia. In addition, all AD patients had received prescriptions of acetylcholinesterase inhibitors suggested by the professional neurologists and psychiatrists in the Bureau of National Health Insurance Committee in Taiwan. Control samples were from subjects who have no signs of severe maladies (i.e., cancer and dementia). Routine clinical chemistry data including alanine transaminase (ALT), creatinine, fasting glucose, glycohemoglobin A1 (HbA1c), high-density lipoprotein cholesterol (HDL-C), low-density lipoprotein cholesterol (LDL-C), total cholesterol, total protein and triglycerides were provided by the Biobank. Referring to the high correlation of AD with MetS, we further divided both control and AD groups based on the MetS-related laboratory test results. Samples with either low HDL-C (≤ 40 mg/dL), high fasting triglycerides (≥ 150 mg/dL), or high fasting glucose (≥ l00 mg/dL) were considered MetS-like. Finally, all samples were grouped as follows: healthy control (HC), MetS-like, AD with normal metabolism (AD-N), and AD with MetS-like conditions (AD-M). Storage of all serum samples was maintained at − 80 °C prior to be analyzed in immunoprecipitation, SDS-PAGE, immunoblotting, and enzyme-linked immunosorbent assay (ELISA). This research was performed as per the Code of Ethics of the World Medical Association and approved by Taipei Medical University Joint Institutional Review Board (N201802044).

### Immunoprecipitation

To detect APP, Aß, and acrolein-modified peptides in mouse brain, mouse serum, and human serum, we performed immunoprecipitation. Rabbit anti-C-terminal APP polyclonal antibody was coupled to protein-G agarose beads (sc-2002, Santa Cruz, TX, USA). Then the beads were incubated with pooled and IgG-removed mouse brain lysates or serum (*n* = 4, 2 females and 2 males), or pooled human serum (*n* = 10, 5 females and 5 males) at 4 °C for 2 h. After washed, the beads were applied with 5% acetic acid, pH 2.28 to elute APP/Aß peptides which were then separated by SDS-PAGE in triplication. One set was stained with Coomassie brilliant blue as a loading control, and the other two sets for immunoblotting with either anti-acrolein or anti-APP antibody.

### Immunoblotting

A protein assay was performed to determine total protein concentration in the lysates of neuro-2a cells and mouse brain and serum samples using Bradford reagent (Bio-Rad Laboratories, Inc., CA, USA). The samples with either 45 µg of proteins from the lysates, 5 µl of IgG-removed pooled serum or 5 µl of immunoprecipitated eluants were separated in 10–18% SDS-PAGE and transferred onto PVDF membrane. A prestained protein ladder with weak luminance (Omics Bio, New Taipei, Taiwan) was used as the marker. After washed, the membrane was blocked with 3% BSA in 1 × PBS at room temperature for 1 h. Followed by washing, the membrane was incubated with primary antibody against either acrolein (2H2, SMC-504, StressMarq Biosciences, Bath, UK) or C-terminal APP in 1 × PBS at 4 °C overnight. After adequate washing, the membrane was incubated with a horseradish peroxidase-conjugated secondary antibody at room temperature for 1 h. The membrane was then washed and developed with chemiluminescent substrate (Thermo Scientific) in a luminescence/fluorescence imaging system (ImageQuant Las-4000). The band density on the blots was quantified using UN SCAN-IT gel 6.1 software (Silk Scientific Crop.).

### ELISA

Diluted human serum (1:100) or synthetic Aß peptides were coated on 96-well plate and kept at 4 °C overnight. Followed by washing, the plate was blocked with 3% BSA (Sigma-Aldrich Co., St. Louis, MO, USA) at 37 °C for 1 h. After blocking, the plate was incubated with either rabbit anti-C-terminal APP, mouse anti-acrolein or human serum (for autoantibody assay) at 4 °C overnight. The plate was washed again and incubated with a horseradish peroxidase-conjugated secondary antibody at 37 °C for 1 h. After washing and drying completely, the plate was added with 50 µL of Microwell Peroxidase Substrate A and B (Kirkegaard and Perry Laboratories, Gaithersburg, MD, USA) each in each well and incubated in the dark at 25 °C for 15 min. The reaction was stopped using 1N HCl. Absorbance was recorded at 450 nm. The values were normalized with the mean in control and shown as relative levels.

### Modification and determination of acrolein on synthetic Aß peptides

Acrolein is also named propylene aldehyde, 2-propenal, acrylaldehyde, and aqualine (Fig. S1). Acrolein-reactive epitopes, including lysine and histidine, are all located at the first 28 amino acid residues on the Aß peptide [25, 26]. Synthetic Aß peptides were designed as Aß_1-16_ and Aß_17-28_ mimicking trypsin-digested products and purchased from Yao-Hong Biotechnology Inc. (New Taipei City, Taiwan). Modifications of acrolein on the synthetic Aß peptides were carried out according to the described protocols [27]. The acrolein-modified peptides were analyzed by injection into nano-LC − ESI–MS/MS analysis and performed on a nano-Acquity system (Waters, Milford, MA, USA) connected to the LTQ-Orbitrap Velos™ hybrid mass spectrometer (Thermo Fisher Scientific, Bremen, Germany) equipped with a PicoViewnanospray interface (New Objective, Woburn, MA, USA) at the Academia Sinica Common Mass Spectrometry Facilities for Proteomics and Protein Modification Analysis. Post-translational modified peptide sequences and sites on the peptides were identified using the PEAKS 7 software (Bioinformatics Solutions Inc., Waterloo, Ontario, Canada). These synthetic peptides with and without acrolein modification were used for autoantibody analyses using ELISA.

### Statistical analysis

SigmaPlot 14 (Systat Software Inc., San Jose, CA, USA) was used for all statistical analyses. Data are presented as the mean ± standard deviation. Student’s *t*-test and one-way ANOVA were performed to determine the statistical significance. When the *p* value is < 0.001, the receiver operating characteristic (ROC) curve is plotted with true-positive rate (sensitivity) against false-positive rate (1 – specificity), and then the area under curve (AUC) was calculated to evaluate the diagnostic performance. The sensitivity of a test was calculated as positive diseased subjects / total diseased subjects. The value of “1 – specificity” was calculated as positive non-diseased subjects / total non-diseased subjects. Pearson product moment correlation was used in correlation analyses. The *p* value of the correlation is the probability of being wrong in concluding that there is a true association between the variables. For all statistical tests, the significant level was set at *p* < 0.05 (*), *p* < 0.01 (**), and *p* < 0.001 (***).

## Results

### Enhanced acrolein adducts on Aß-containing peptides in AD model cells and mice

**Fig. 1:**
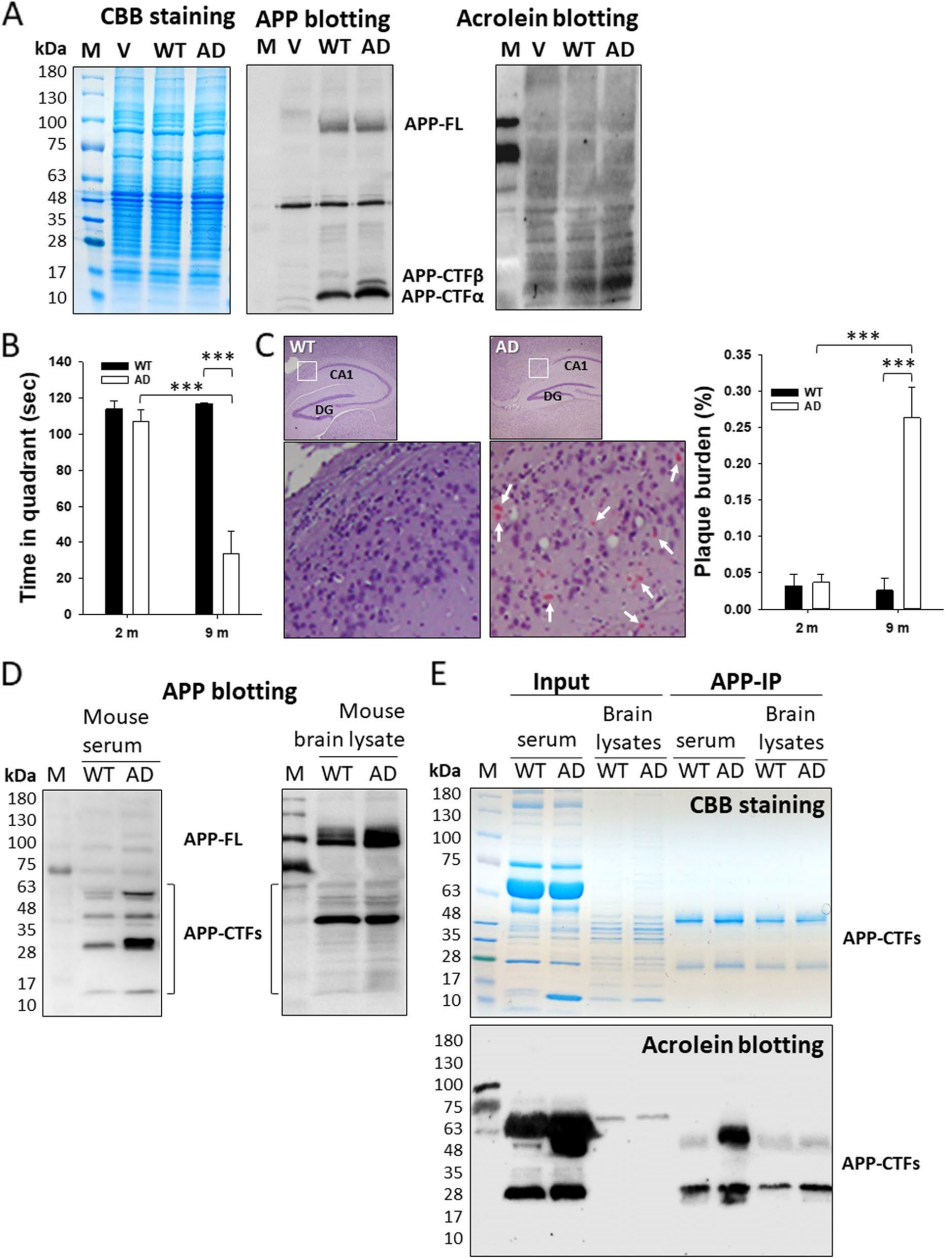
Acrolein adducts in the transfected cells and model mouse tissues. **A** Immunoblotting of the cell lysates. The transfected neuro-2a cells were analyzed with SDS-PAGE in triplicate. One gel was stained with CBB as a loading control. The other two gels were immunoblotted with anti-C-terminal APP and acrolein antibodies, respectively. The full-length APP (APP-FL) and APP C-terminal fragments (APP-CTFa and APP-CTFß) were indicated. **B** Morris Water Maze analyses. The time of the mice (*n* = 9) spent in the target quadrant at 2 and 9 months old was recorded and compared. **C** Congo red staining of Aß plaques in the mouse brain. Aß plaques in the cortex and hippocampus of the mice (*n* = 6) were stained. The plaque burden was quantified and compared. **D** APP immunoblotting of mouse serum and brain lysates. The IgG-removed serum and brain tissues of the mice at 9 months old were immunoblotted with anti-C-terminal APP antibody, and the gel stained with CBB was used as a loading control. The full-length APP (APP-FL), APP C-terminal fragments (APP-CTFs), and Aß oligomers were indicated. **E** APP immunoprecipitation and acrolein immunoblotting of mouse serum and brain lysates. The IgG-removed serum and brain tissues of the mice at 9 months old were immunoprecipitated with anti-C-terminal APP antibody and then subjected to immunoblotting with anti-acrolein antibody. A duplicated gel was stained with CBB as a loading control. The prestained protein ladder showed some luminance. AD, Alzheimer’s disease; APP, amyloid precursor protein; CBB, Coomassie brilliant blue; CTF, C-terminal fragment; IP, immunoprecipitation; M, marker; V, vector plasmid p-CAX; WT, wild-type. *** *p* < 0.001

Cellular disturbance in AD pathology has been demonstrated in the AD model cells [20, 21]. APP-CTFs were intermediates of APP processing from APP-FL. APP-CTFß is from the cleavage by ß-secretase, representing the amyloidogenic activity in the cells. The level of APP-CTFs, especially APP-CTFß, in the AD model cells, was increased when referred to the level of APP-FL and compared to that in WT (Fig. 1A, middle panel). These results are consistent with the previous observation that amyloidogenic processing is increased in the diseased cells [20, 21]. Notably, when acrolein was immunoblotted, a higher level of reactive bands was seen in AD model cells than in WT cells, especially in the low molecular weight region overlapping APP-CTFs (Fig. 1A, right panel). The bands looked blurry possibly due to long exposure in image development. It suggests that the AD cells may undergo metabolic disturbance with increased acrolein adducts probably formed on Aß peptides.

To investigate the acrolein adduction in an animal model, we used 3xTg-AD mice and tested them using Morris water maze, histochemistry, immunoprecipitation, and immunoblotting. The water maze test revealed that 3xTg-AD mice spent a shorter time in the target quadrant than did WT with a statistical significance at 9 months old (*p* < 0.001) (Fig. 1B). Congo red-stained mouse brain sections showed an increased Aß plaque burden in AD mice compared to WT mice at 9 months old, especially in the cortical and hippocampal regions (Fig. 1C). Immunoblotting showed higher band intensity of APP isoforms and fragments including APP-CTFs which contain Aß peptides in the serum and brain lysates of the AD mice compared to WT mice (Fig. 1D). After the AD mouse model was verified, serum and brain lysates of the mice were used to analyze acrolein adduction. Pooled mouse serum and brain lysates were immunoprecipitated with a C-terminal APP antibody and then immunoblotted with acrolein. The level of acrolein adducts was increased obviously in the pooled AD mouse serum and brain lysates compared to those in WT mice, especially on APP-CTFs (Fig. 1E). These results further demonstrated the specific acrolein modification on Aß-containing peptides.

### Correlation of acrolein adducts with MetS in AD patients

**Fig. 2:**
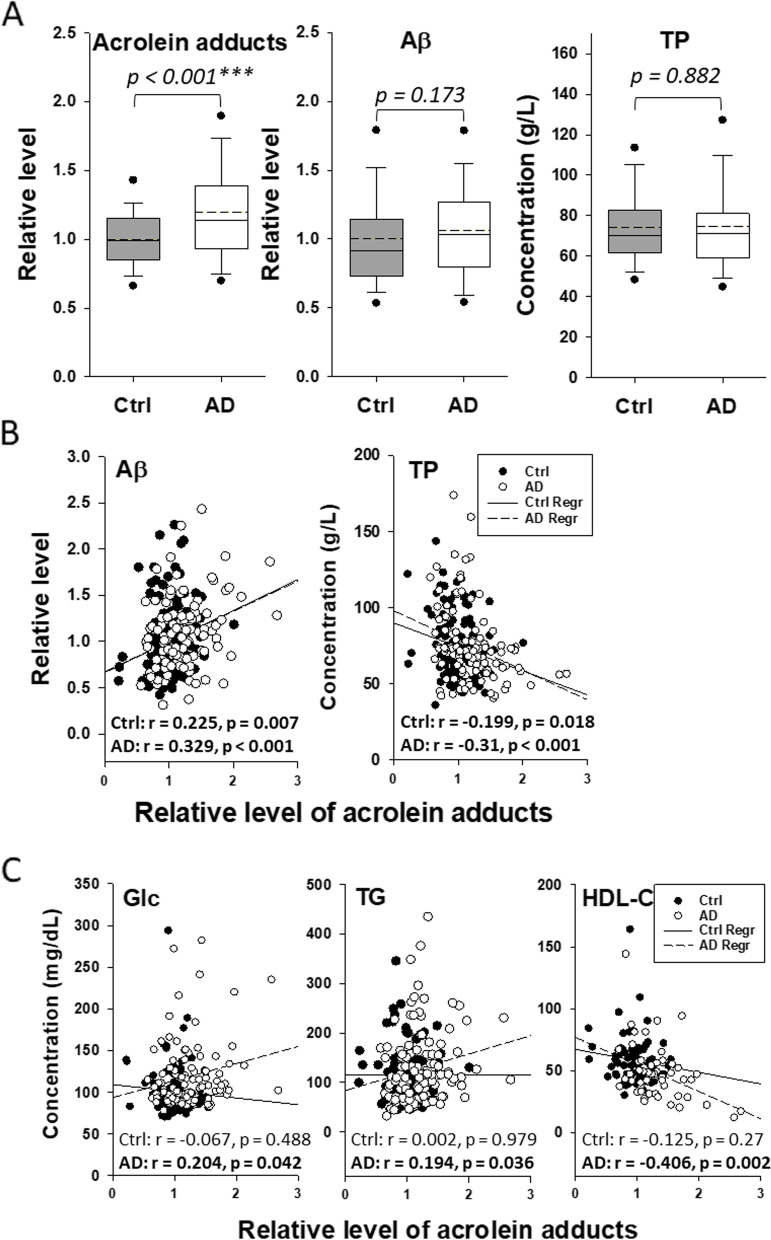
Correlation of acrolein adducts with specific metabolites in human serum. **A** Levels of Aß, acrolein adducts, and total protein (TP) in human serum samples. All the values were normalized with the mean of control. In the box plots, the dots represent the 5th and 95th percentiles; the error bars cover the 10th to 90th percentiles and the box covers the 25th to 75th percentiles; the solid and dash lines within the box represent the median and mean values, respectively. **B** Correlation of acrolein adducts with Aß and TP. Serum Aß and TP values were plotted against the level of acrolein adducts in control and AD subjects, respectively. The solid and broken lines represent the linear regression of the values in control and AD subjects, respectively. Pearson product moment correlation coefficients (r) of acrolein adducts with Aß and TP were shown with *p* values. **C**. Correlation of acrolein adducts with the metabolites related to metabolic syndrome. Serum fasting glucose (Glc), triglycerides (TG), and HDL-C values of control or AD subjects were plotted against the level of acrolein adducts. The solid and broken lines represent the regression of the values in control and AD subjects, respectively. Pearson product moment correlation coefficients (r) of acrolein adducts with Glc, TG, and HDL-C were shown with *p* values. Aß, amyloid-beta; AD, Alzheimer’s disease; Ctrl, control; HDL-C, high-density lipoprotein-cholesterol; TG, triglycerides; TP, total protein.*** *p* < 0.001

The levels of Aß and acrolein adducts in human serum were measured. The concentrations of biochemicals in routine clinical tests were provided by the Biobank as described in Materials and Methods. As shown in Fig. 2, the average levels of Aß and total protein seemed unchanged in the serum of AD patients compared to those of the control subjects, whereas acrolein adducts were significantly increased in AD serum (*p* < 0.001) (Fig. 2A). Interestingly, Pearson correlation analysis revealed that the level of acrolein adducts correlated positively with Aß and negatively with total protein (Fig. 2B). It suggests that acrolein adducts may be largely formed on Aß, which supports the findings in the AD models (Fig. 1A and E). Furthermore, the negative correlation of acrolein adducts with total protein in human serum may indicate a reduction of certain types of protein (i.e., antibodies) when acrolein adducts were increased in AD blood. Notably, significant correlations were also found between acrolein adducts and MetS parameters, including fasting glucose, triglycerides, and HDL-C (Fig. 2C). Therefore, both control and AD groups were further divided according to MetS-related laboratory test results as described in the “Materials and methods” section. However, the average age of the AD subjects was unexpectedly older than the control subjects with statistical significance. Therefore, Pearson correlation analysis was also performed to clarify the effects of age on these parameters, especially acrolein adducts, in the AD and non-AD control groups. It was plausible that age was not correlated with acrolein adducts or most of the tested parameters, except the negative correlation of age with total cholesterol and HDL-C in the control group (Table S1).

Clinical profile and laboratory test results of the four groups, namely HC, MetS, AD-N, and AD-M, were summarized with mean ± SD in Table 1 and *p* values in Table S2. As expected, significant differences in fasting glucose, triglycerides, and HDL-C were observed between the groups with and without metabolic disturbance due to the sorting criteria. Likewise, HbA1c, LDL-C, and creatinine were also significantly different between the groups owing to MetS-related conditions. Although the average concentrations of both HDL-C and LDL-C were significantly lower in AD-M than in the MetS group, they were not considered different because of the age effect as mentioned (Table S1). No statistical differences were seen in gender, ALT, total cholesterol, and total protein between the groups.

**Table 1:** Demographic and clinical profile of the groups (mean ± SD)

	**HC** (***n* = 84**)	**MetS** (***n* = 58**)	**AD-N** (***n* = 34**)	**AD-M** (***n* = 83**)	**Reference interval**^**a**^
***Gender (female:male)***	45:39	21:37	17:17	43:40	-
***Age (year)***	74.8 ± 4.9	76.3 ± 5.8	81.3 ± 5.4	82.0 ± 6.8	-
***ALT (IU/L)***	21 ± 15	27 ± 17	17 ± 7	23 ± 18	< 41
***Creatinine (mg/dL)***	1.0 ± 0.4	1.0 ± 0.5	1.0 ± 0.3	**1.5**^b^ ± 1.4	0.6—1.2 (53 – 106 µmol/L)
***Fasting glucose (mg/dL)***	87 ± 9	**115 **^b^ ± 34	92 ± 6	**128**^b^ ± 42	70 – 99 (3.9 – 5.5 mmol/L)
***HbA1c (%)***	5.7 ± 0.1	**6.4 **^b^ ± 1.1	5.6 ± 0.3	**6.3**^b^ ± 0.9	4—6
***HDL-C (mg/dL)***	62 ± 22	55 ± 16	70 ± 27	41 ± 15	> 40 (> 1.04 mmol/L)
***LDL-C (mg/dL)***	107 ± 27	118 ± 32	111 ± 24	100 ± 29	< 130 (< 3.37 mmol/L)
***Total cholesterol (mg/dL)***	171 ± 37	190 ± 37	188 ± 34	177 ± 42	< 200 (< 5.18 mmol/L)
***Total protein (g/L)***	72 ± 17	78 ± 23	73 ± 20	75 ± 26	66 – 87
***Triglycerides (mg/dL)***	101 ± 24	135 ± 60	88 ± 31	145 ± 79	< 150 (< 1.69 mmol/L)

*AD-M* Alzheimer’s disease with metabolic syndrome, *AD-N* Alzheimer’s disease with normal metabolism, *HbA1c* glycohemoglobin A1, *HC* healthy control, *HDL-C* high-density lipoprotein-cholesterol, *LDL-C* low-density lipoprotein-cholesterol, *MetS* metabolic syndrome

^a^The International System of Units (SI) of reference interval were shown in brackets

^b^The mean values exceeding reference intervals were shown in bold

### Enhanced acrolein-Aß adducts in the serum of AD patients with metabolic disturbance

**Fig. 3:**
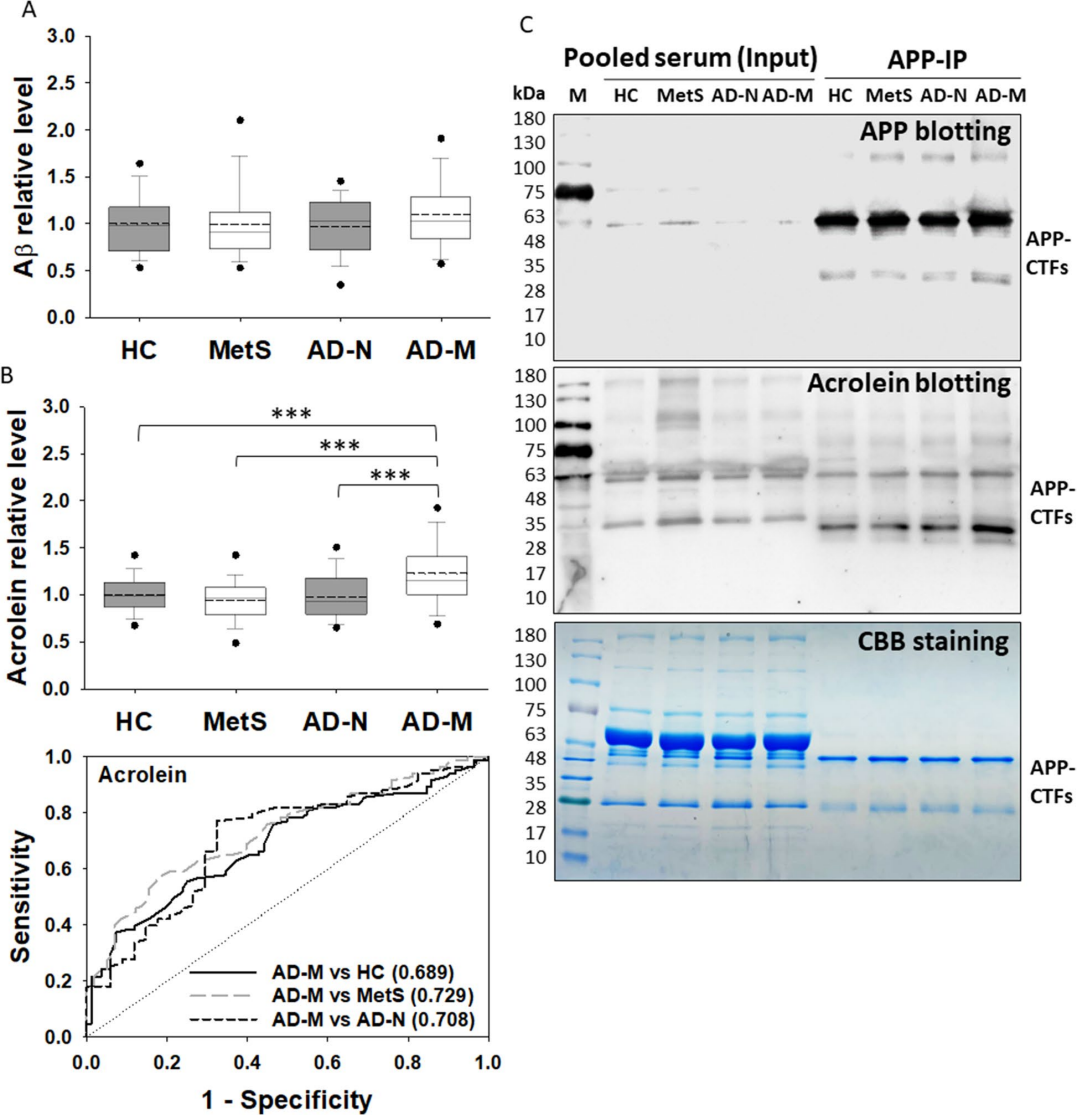
Acrolein adducts on APP fragments and Aß peptides in human serum. **A** The relative levels of Aß in different groups of human serum sample. **B** The relative levels of acrolein adducts in different groups of human serum sample. All the values were normalized with the mean of healthy control (HC). The dots represent the 5th and 95th percentiles; the error bars cover the 10th to 90th percentiles and the box covers the 25th to 75th percentiles; the solid and dash lines within the box represent the median and mean values, respectively. The receiver operating characteristic curves were plotted with the area under curve values shown in brackets when the *p* value is < 0.001 between groups. **C** APP immunoprecipitation and acrolein immunoblotting of human serum. Pooled human serum samples from each group were IgG-removed and immunoprecipitated with anti-C-terminal APP antibody and then subjected to SDS-PAGE in triplicate. Two gels were immunoblotted with anti-C-terminal APP and acrolein antibodies, respectively. Another gel was stained with CBB as a loading control. The prestained protein ladder showed some luminance. Aß, amyloid beta; AD-M, Alzheimer’s disease with metabolic disturbance; AD-N, Alzheimer’s disease with normal metabolism; APP, amyloid precursor protein; CBB, Coomassie brilliant blue; HC, healthy control; IP, immunoprecipitation; M, marker; MetS, metabolic syndrome.***p < 0.001

Among the four groups, no significant difference in Aß level was observed (Fig. 3A). There were no significant differences in acrolein adducts observed among HC, MetS, and AD-N groups, either (Fig. 3B). Only in the AD-M group, the level of acrolein adducts was significantly increased compared to all other groups. The best AUC value of ROC analysis on acrolein adducts was observed in distinguishing AD-M from MetS (AUC = 0.729). These results suggest that acrolein adducts may be a better biomarker than Aß for not only AD pathology but also the pathogenesis from metabolic disturbance to neurodegeneration in AD.

To detect acrolein adducts in more detail, human serum samples were pooled and subjected to immunoprecipitation and immunoblotting. The major APP species appeared near 48 kDa, and a minor species was seen near 28 kDa in the serum samples (Fig. 3C). They have been suggested as truncated APP-CTFs [28]. The low molecular-weight fragments were highly reactive to anti-acrolein antibody, especially in AD-M samples. These results were consistent with the findings in AD models and the ELISA data from human subjects, indicating that the formation of acrolein-Aß adducts may be highly correlated with metabolic disturbance in AD pathogenesis.

### In vitro acrolein-modified Aß analyses

**Fig. 4:**
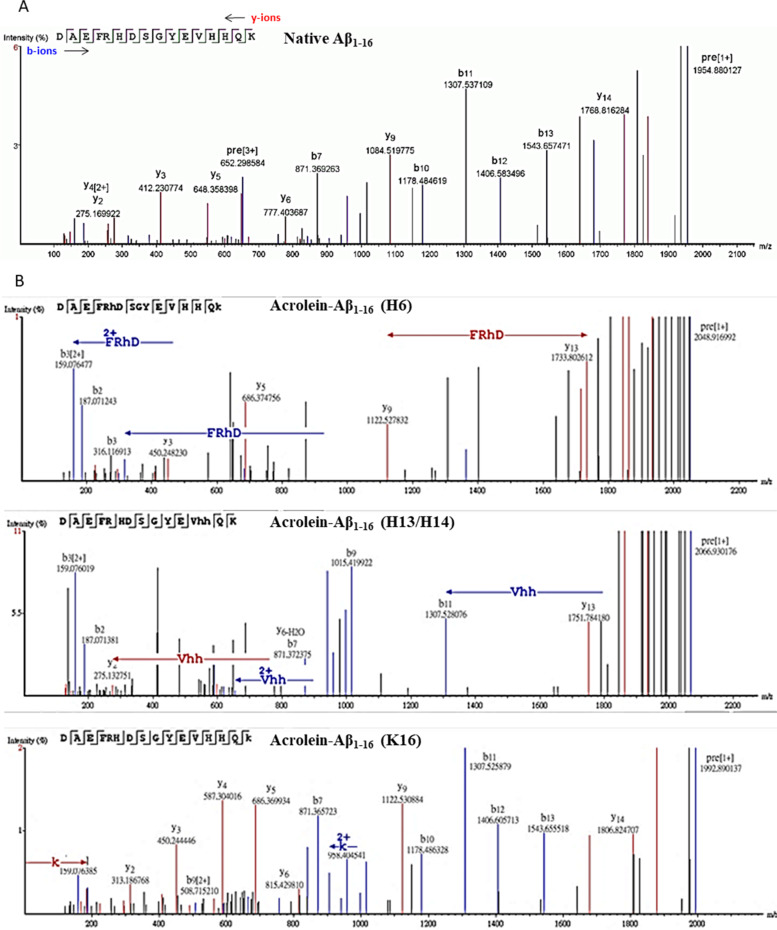
Mass spectra of the native and acrolein-modified Aß_1-16_. The ion density (*y*-axis) was plotted against m/z ratio (*x*-axis). The b-ions (blue peaks) represent N-terminal fragments, whereas y-ions (red peaks) represents C-terminal fragments. Acrolein-modified histidine and lysine were pointed with arrows. **A** Native Aß_1-16_ peptide. **B** Acrolein-modified Aß_1-16_ at H6, H13, H14 or K16. Only the selected spectra were shown

**Fig. 5:**
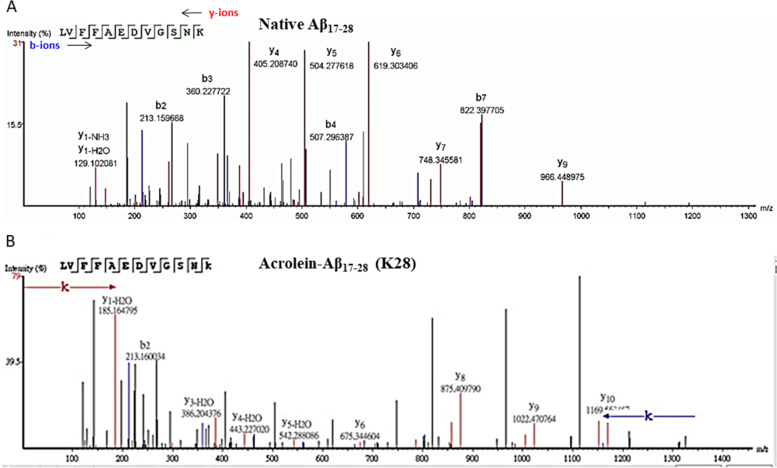
Mass spectra of the native and acrolein-modified Aß_17-28_. The ion density (*y*-axis) was plotted against m/z ratio (*x*-axis). The b-ions (blue peaks) represents N-terminal fragments, whereas y-ions (red peaks) represents C-terminal fragments. Acrolein-modified histidine and lysine were pointed with arrows. **A** Native Aß_17-28_ peptide. **B** Acrolein-modified Aß_17-28_ at K28. Only the selected spectra were shown

To further analyze acrolein-Aß adducts and their responding autoantibodies, we used synthetic Aß_1-16_ and Aß_17-28_ peptides and modified them with acrolein in vitro. Acrolein modification of Aß was verified by LC–MS/MS. Native and modified peptides were identified as b-ion (N-terminal fragments) and y-ion (C-terminal fragments) series. Native Aß_1-16_ fragment ion showed the [M + H]^+^ at 1953.87 and Aß_17-28_ at 1324.67 (Figs. 4A and 5A and Table 2). The molecular weight of acrolein is 56.06 Da. When acrolein binds proteins, four different types of adduct including schiff-base (+ 38 Da), aza-michael (+ 56 Da), Ne-lysine (MP-lysine) (+ 76 Da), and FDP-lysine (+ 94 Da) can be formed [29]. Acrolein modification on synthetic Aß peptides were observed on H6, H13, H14, and K16 on Aß_1-16_, and K28 on Aß_17-28_ (Figs. 4B and 5B and Table 2). Mainly aza-michael adducts were found on histidine residues, while all four types of adduct were found on lysine residues with schiff-base in the majority. The highest intensity of acrolein adducts was seen on K28 (Fig. 5B), while the most spectral count of the adducts was on K16 (Table 2). These results were consistent with the finding of acrolein modification on Aß-containing peptides as seen in mouse serum and brain lysates (Fig. 1) and human serum (Fig. 3).

**Table 2:** Aß peptides with acrolein-modified residues

Peptide	-10lgP	Mass	ppm	m/z	z	RT	Scan	#Spec
**Aß1-16**	113.90	1953.87	- 2.2	652.2964	3	18.53	996	67
**DAEFRh(+ 56.03)DSGYEVHHQK**	113.72	2009.90	2	503.48	4	18.25	961	8
**AEFRHDSGYEVh(+ 56.03)HQK**	114.37	1894.87	1.1	474.73	4	18.02	934	1
**DAEFRHDSGYEVh(+ 56.03)HQK**	103.57	2009.90	1.3	670.97	3	19.83	1142	8
**AEFRHDSGYEVHh(+ 56.03)QK**	99.88	1894.87	1.5	379.98	5	18.09	943	1
**DAEFRHDSGYEVHh(+ 56.03)QK**	49.81	2009.90	1.1	402.99	5	51.7	3060	1
**AEFRHDSGYEVHHQk(+ 38.02)**	123.47	1876.86	0.9	470.22	4	18.11	945	2
**DAEFRHDSGYEVHHQk(+ 38.02)**	147.15	1991.89	4.8	664.97	3	21.56	1309	14
**DAEFRHDSGYEVHHQk(+ 76.03)**	58.39	2029.90	0.5	508.48	4	19.77	1135	1
**DAEFRHDSGYEVHHQk(+ 94.04)**	105.66	2047.91	2	512.99	4	18.59	1014	6
**DAEFRh(+ 56.03)DSGYEVh(+ 56.03)HQK**	70.98	2065.92	0.8	517.49	4	18.46	995	2
**DAEFRh(+ 56.03)DSGYEVHHQk(+ 38.02)**	58.64	2047.91	2.6	512.99	4	20.67	1225	1
**DAEFRHDSGYEVh(+ 56.03)h(+ 56.03)QK**	53.00	2065.92	4	414.19	5	19.46	1108	3
**DAEFRHDSGYEVh(+ 56.03)HQk(+ 38.02)**	68.40	2047.91	- 1	512.99	4	21.79	1331	2
**DAEFRHDSGYEVHh(+ 56.03)Qk(+ 94.04)**	81.45	2103.94	1.1	526.99	4	19.92	1150	3
**Aß17-28**	78.80	1324.67	1.5	442.5634	3	29.91	1610	22
**LVFFAEDVGSNk(+ 38.02)**	41.34	1362.69	0.8	682.35	2	29.8	1589	2
**LVFFAEDVGSNk(+ 56.03)**	27.51	1380.70	0.5	691.35	2	30.76	1669	1
**LVFFAEDVGSNk(+ 76.03)**	76.84	1400.70	0.8	701.36	2	30.74	1667	1
**LVFFAEDVGSNk(+ 94.04)**	60.88	1418.71	0.4	710.36	2	30.41	1644	2

*Aß* amyloid-beta, *ppm* part per million (mass error), *RT* retention time (min)

### Alterations in anti-acrolein autoantibodies in AD patients with metabolic disturbance

Pearson correlation analysis revealed a negative correlation of acrolein adducts with total protein, especially in AD blood (Fig. 2B). We hypothesize that the increase of acrolein adducts as seen in AD-M condition (Fig. 3B) may be due to the depletion of acrolein-responding autoantibodies which are supposed to be an abundant protein type to neutralize toxic acrolein adducts. To test this hypothesis, we measured anti-acrolein antibody isotypes IgG and IgM in the human serum samples using acrolein-modified synthetic Aß_1-16_ and Aß_17-28_ peptides as antigens in ELISA. Native Aß peptides were used as control items.

**Fig. 6:**
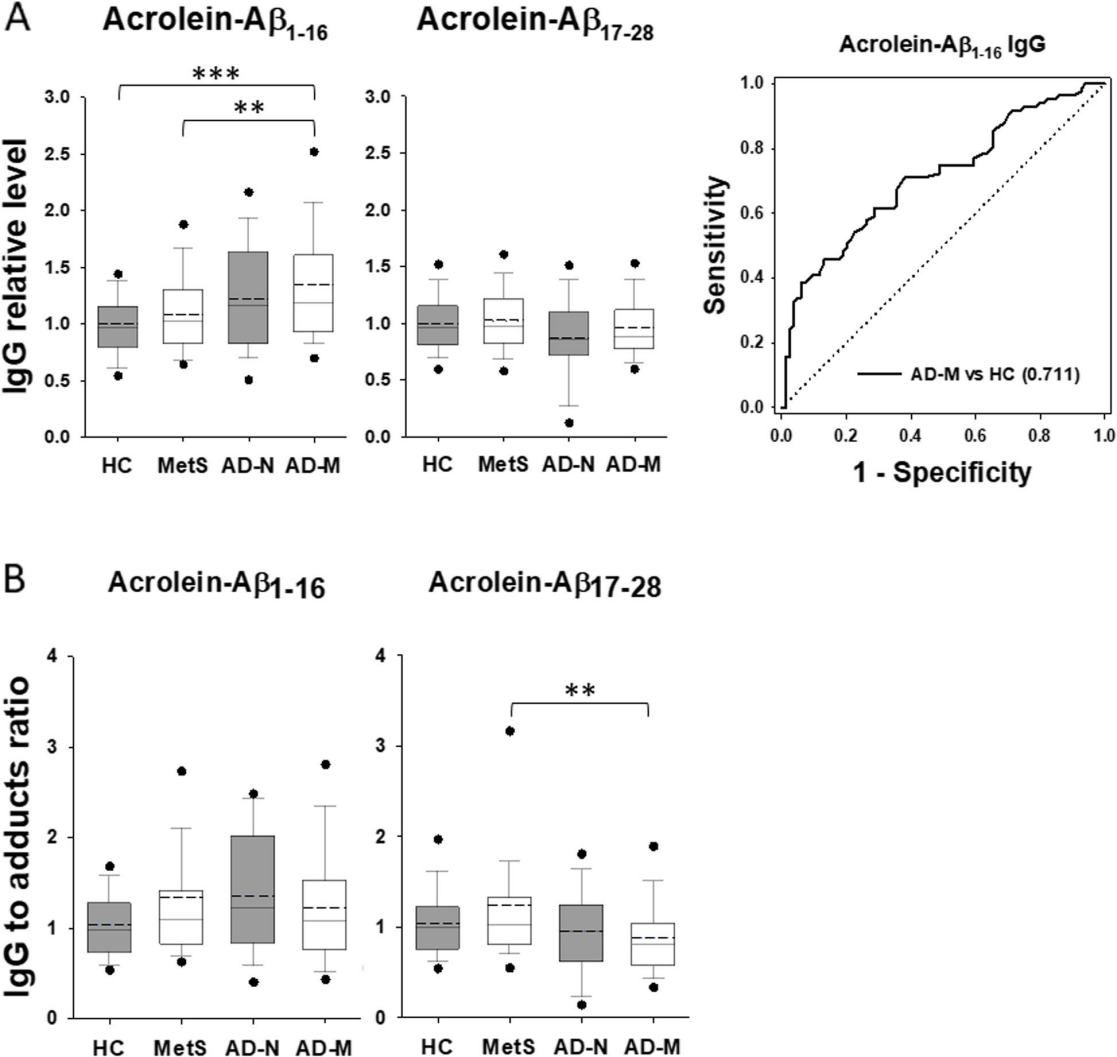
Change of IgG autoantibodies against acrolein-Aß adducts in human serum. **A** The relative levels of IgG autoantibody recognizing acrolein-modified Aß_1-16_ and Aß_17-28_ peptides in each group. All the values were normalized with the mean of healthy control (HC). **B** The ratio of the responding IgG to acrolein adducts of each subject in each group. In the box plots, the dots represent the 5th and 95th percentiles. The error bars cover the 10th to 90th percentiles and the box covers the 25th to 75th percentiles. The solid and dashed lines within the box represent the median and mean values, respectively. The receiver operating characteristic curves were plotted with the area under curve values shown in brackets when the *p* value is < 0.001 between groups. Aß, amyloid beta; AD-M, Alzheimer’s disease with metabolic disturbance; AD-N, Alzheimer’s disease with normal metabolism; HC, healthy control; MetS, metabolic syndrome.**p < 0.01, ***p < 0.001

No differences were seen in the relative levels of IgG against native Aß peptides among all groups (Fig. S2A). However, the ratio of anti-Aß_1-16_ IgG to the peptide was reduced slightly in the AD-M group compared to HC although the ratio of anti-Aß_17-28_ IgG to the peptide remained no difference among all groups (Fig. S2B). Unlikely, the relative level of IgG against acrolein-modified Aß_1-16_ was increased in the AD-M group moderately when compared to MetS and largely when compared to HC (*p* < 0.001) with an AUC value of 0.711 in ROC analysis (Fig. 6A). Nevertheless, the level of IgG against acrolein-modified Aß_17-28_ showed the same among all groups. Notably, the ratio of anti-acrolein-Aß_1-16_ IgG to acrolein adducts became no difference among all groups, while the ratio of anti-acrolein-Aß_17-28_ IgG to acrolein adducts was reduced moderately in AD-M group compared to MetS (Fig. 6B). Since the immune responses shall depend on the antigen levels, the ratio of the antibody to the specific antigen may provide a more useful evaluation. Therefore, these results suggest that the responding IgG levels, despite anti-native Aß or anti-acrolein-modified Aß, were reduced with slight to moderate amounts when MetS patients developed AD.

**Fig. 7:**
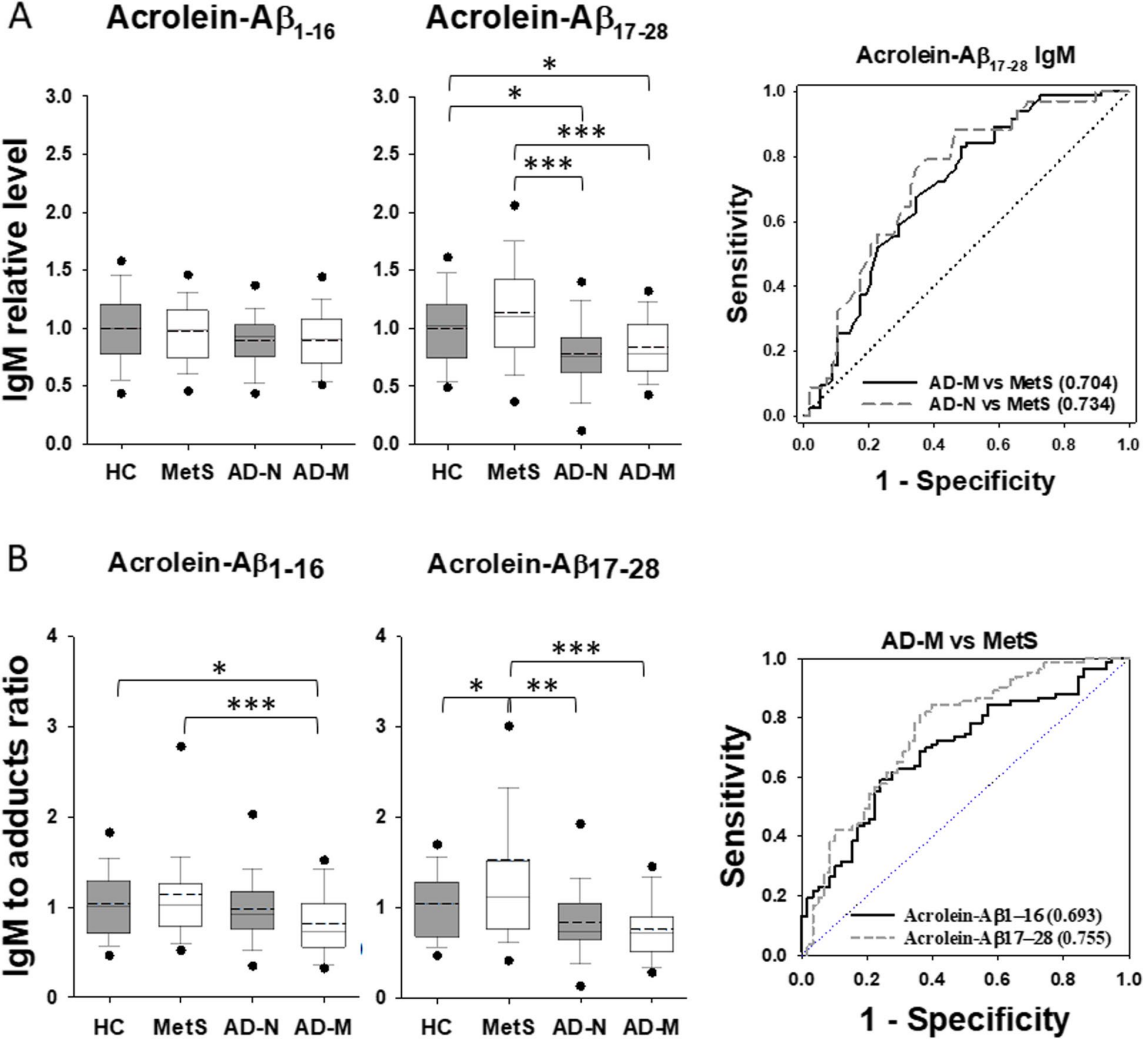
Change of IgM autoantibodies against acrolein-Aß adducts in human serum. **A** The relative levels of IgM autoantibody recognizing acrolein-modified Aß_1-16_ and Aß_17-28_ peptides in each group. All the values were normalized with the mean of healthy control (HC). **B** The ratio of the responding IgM to acrolein adducts of each subject in each group. In the box plots, the dots represent the 5th and 95th percentiles. The error bars cover the 10th to 90th percentiles and the box covers the 25th to 75th percentiles. The solid and dashed lines within the box represent the median and mean values, respectively. The receiver operating characteristic curves were plotted with the area under curve values shown in brackets when the *p* value is < 0.001 between groups. Aß, amyloid beta; AD-M, Alzheimer’s disease with metabolic disturbance; AD-N, Alzheimer’s disease with normal metabolism; HC, healthy control; MetS, metabolic syndrome.* *p* < 0.05, **p < 0.01, ***p < 0.001

Similarly, both the level of IgM against native Aß_1-16_ and the ratio of anti-Aß_1-16_ IgM to the peptide were reduced moderately in the AD-M group compared to both MetS and HC (Fig. S3). In contrast, the level of IgM against acrolein-Aß_17-28_ and the ratio of both anti-acrolein-Aß_1-16_ and anti-acrolein-Aß_17-28_ IgM antibodies to acrolein adducts were reduced largely in AD-M group compared to MetS (*p* < 0.001) with AUC values from 0.693 to 0.755 in ROC analyses (Fig. 7). It was supportive in Pearson correlation analyses that the level of acrolein adducts was negatively correlated with anti-acrolein-Aß_17-28_ IgM although it was positively correlated with the specific IgG against native Aß_1-16_ in all samples (Table S3). These results suggest that the specific IgM autoantibodies against acrolein adducts, such as acrolein-Aß, were reduced largely when MetS patients developed AD and are potential biomarkers in AD pathology. In addition, acrolein-Aß adducts may be considered for the immunotherapy of AD, especially when AD is complexed with MetS.

## Discussion

A recent Korean study demonstrates the association between MetS to developed AD in eight years [6]. It suggests that MetS patients are 11.48 times more prone to have AD than those without MetS. Statistical analysis of Taiwanese primary data from the National Health Insurance Research Database with encryption also reports the link between AD progressions from MetS [5]. This study explained the statistically significant relationship between dementia and worsen MetS compared to non-persistent MetS, improved MetS, or non-MetS in the follow-up period of 10 years. MetS is a prolonged state of oxidative stress which is resulted from abnormal metabolism of carbohydrates and lipids, inducing lipid peroxidation to generate aldehydic byproducts, especially acrolein adducts. Along with the accumulation of Aß in the diseased brain, the immune system may be stimulated to worsen AD progression.

In this study, acrolein adducts were found to correlate positively with triglycerides and negatively with HDL-C (Fig. 2C and Table S3). Research suggests that precautionary measurement in early (35–50 years) to middle (51–60 years) adulthood to maintain HDL-C and triglycerides levels may lower the risks of AD [30]. High circulating HDL levels have been linked to a lower risk of Alzheimer's disease in numerous epidemiological studies [24]. HDL appears to protect against memory loss, neuroinflammation, and cerebral amyloid angiopathy by lowering vascular Aß accumulation and attenuating Aß-induced endothelial inflammation. In contrast, high level of triglycerides can cause cognitive decline by blood brain barrier (BBB) dysfunction or amyloid metabolism imbalance [31]. However, cognitive function can be improved by coconut oil or medium-chain triglycerides. Omega-3 polyunsaturated fatty acids (ω-3-PUFAs) are key components of the neuronal membrane which acts as neuroprotective agent [32]. Impaired neurotransmission was observed in ω-3-PUFA-deficient animal [33]. Reduction in Aß production by altering APP pathological processing pathway is due to docosahexaenoic acid (22:6ω-3). A negative correlation was found in ω-3/ω-6 ratio with AD incidence and cognitive decline. Reduced docosahexaenoic acid and eicosapentaenoic acid (20:5ω-3) levels have been observed in AD brain autopsy, serum, and plasma.

Byproducts of PUFA oxidation are acrolein and HNE specifically from ω-6 PUFAs, such as arachidonic acid which is abundant in gray matter [2, 34]. Arachidonic acid oxidation in the presence of Aß in vitro produces HNE earlier than acrolein. It causes a high level of HNE at first and then leads to increased HNE-protein adducts but little extractable free HNE. In contrast, fewer acrolein-protein adducts and more extractable acrolein is produced. It has been shown that acrolein is much more toxic than HNE as seen in the primary culture of rat hippocampal neurons [7]. Likewise, we observed less level of overall acrolein adducts than HNE adducts in human serum using immunoblotting (Fig. 3C) [18]. Interestingly, in the APP immunoprecipitated materials from brain lysates of mouse and serum of mouse and human, acrolein adducts were formed more on the lower molecular weight (~ 28 kDa) than on the higher molecular weight (~ 48 kDa) APP-CTFs (Figs. 1E and 3C), while HNE adducts in human serum are formed in the opposite way [18]. As per our conclusion, acrolein-Aß adducts may play a central role in AD pathology and could be a better biomarker than HNE adducts and Aß alone in AD serum.

The present data showed significantly decreased levels of specific autoantibodies, especially IgM, in AD-M compared to the MetS group (Figs. 6 and 7, S2, and S3). It suggests that IgM is highly involved in the pathogenesis from MetS to AD. IgM plays important roles in the recognition of acrolein-specific epitopes to trigger autoimmunity [35]. The immunological imbalance may increase Aß production or Aß clearance. A high level of IgM indicates a recent infection, whereas a low level represents a continuous existence of the pathogen or a harmful substance. The increased level of acrolein-Aß adducts in AD conditions, especially AD-M (Fig. 4), is negatively correlated with the level of IgM against these adducts (Table S3). It indicates that the toxicities of acrolein and Aß are neutralized by the immune response in a pre-AD condition such as MetS. When acrolein and Aß keep produced, they form adducts and deplete or inhibit the responding antibodies, resulting in the development of neurodegeneration in AD.

Immunotherapy targeting Aß has been approved for AD treatment although the outcome is still doubtable [36]. The domain on Aß for targeting could be a concern. A study reveals that autoantibodies to Aß are epitope dependent in AD [17]. The levels of autoantibody targeting Aß_1-18_ increased and those targeting the Aß_19-36_ decreased. However, our study showed no significant change of most autoantibodies targeting native Aß peptides, except a slight decrease of IgM targeting Aß_1-16_ in AD instead (Fig. S2). Perhaps different protocols and the disease stages contributed to the inconsistency on the analysis of native Aß. Interestingly, the level of autoantibodies targeting acrolein-modified Aß peptides changed significantly in AD patients (Figs. 6 and 7). Thus, we propose that acrolein-Aß adducts and the responding antibodies may be better targets than native forms of Aß for diagnosis and treatment of AD.

Acrolein can be generated exogenously (smoke of cigarette, overheated cooking oil, or fuel combustion) or endogenously (from amino acid oxidation, polyamine metabolism or lipid peroxidation) [10, 37]. Polyamine like spermine helps for the suppression of Aß aggregation. A high level of acrolein forms conjugation with substrate and catalyst involved in tricarboxylic acid cycle, glycolysis, and carbon metabolism. These conjugations inhibit the energy metabolism under oxidative stress in AD conditions. Among acrolein-related risk factors for AD, smoking is also important due to increased oxidative stress and specially acrolein production. Impaired nitric oxide synthesis due to smoking alters glucose metabolism and cerebral blood flow in cerebral vascular endothelial cells of the brain. It stimulates Aß production, cerebral hypoperfusion, cytokine activation, and immune response, which are also helpful for AD diagnosis [38, 39].

## Conclusion

**Fig. 8:**
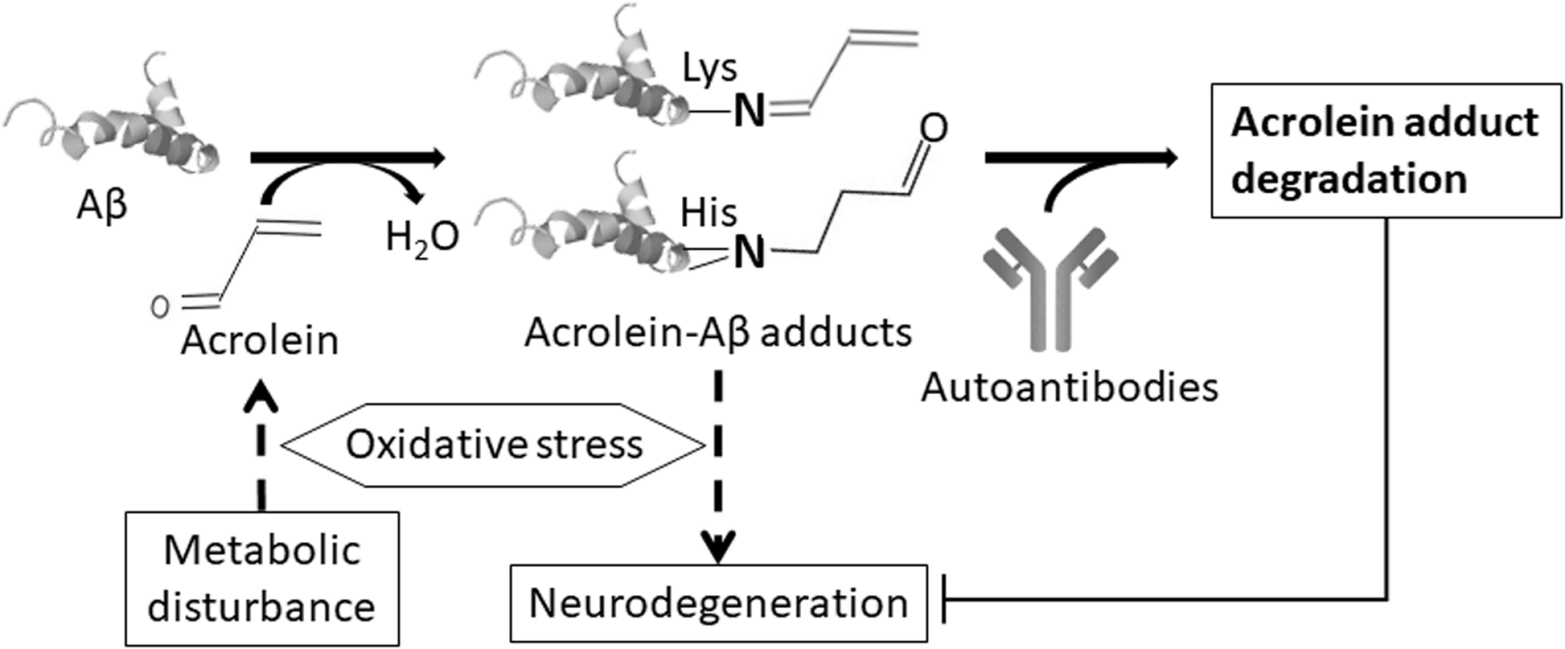
Proposed reactions of acrolein-Aß adduct formation and degradation in correlation with metabolic disturbance and neurodegeneration. Under metabolic disturbance, oxidation of lipids generates aldehydic byproducts such as acrolein, which further reacts with peptides such as Aß and forms adducts. These adducts are responsible for neurodegeneration. However, the responding autoantibodies may inhibit the disease progression by neutralizing and degrading these adducts. Depletion of the responding autoantibodies by acrolein adducts may lead to worse neurodegeneration

Based on the present data, we conclude that metabolic disturbance may induce acrolein production and form adducts largely on Aß through oxidative stress. These adducts may lead to alterations in the autoantibodies, in which an increased level helps to slow disease progression by neutralizing and degrading acrolein adducts, whereas a decreased level of autoantibodies may lead to worse neurodegeneration and cause severe AD. This study explains the close relationship between AD and MetS by suggesting the mechanism that acrolein adduction in MetS may deplete responding autoantibodies and cause neurodegeneration in AD (Fig. 8).

## Supplementary Information

**Additional file 1: Table S1.** Pearson correlation of age with the tested variables. **Table S2.** Statistical significance of demographic and clinical data (p value) between the groups. **Table S3.** Correlation of all tested variables. **Fig. S1.** Acrolein structure. PubChem, Acrolein. National Center for Biotechnology Information, 2022. Retrieved from https://pubchem.ncbi.nlm.nih.gov/compound/Acrolein. **Fig. S2.** Levels of IgG autoantibodies against native Aß peptides in human serum. **Fig. S3.** Levels of IgM autoantibodies against native Aß peptides in human serum.

## Data Availability

Data sharing is not applicable to this article. All other data are available from the corresponding author on reasonable request.

## Abbreviations

Aß: Amyloid-beta
AD: Alzheimer’s disease
AD-M: Alzheimer’s disease with metabolic disturbance
AD-N: Alzheimer’s disease with normal metabolism
APP: Amyloid precursor protein
AUC: Area under curve
BBB: Brain blood barrier
BSA: Bovine serum albumin
CBB: Coomassie brilliant blue
Ctrl: Control
ELISA: Enzyme-linked immunosorbent assay
HbA1c: Glycohemoglobin A1
HC: Healthy control
HDL: High-density lipoprotein-cholesterol
HNE: 4-Hydroxynonenal
LC–MS-MS: Liquid chromatography with tandem mass spectrometry
LDL-C: Low-density lipoprotein-cholesterol
MetS: Metabolic syndrome
PBS: Phosphate buffer saline
PUFA: Polyunsaturated fatty acid
ROC: Receiver operating characteristic
ROS: Reactive oxygen species
SDS-PAGE: Sodium dodecyl sulfate–polyacrylamide gel electrophoresis

## References

[1] Wolfe MS (2003). The secretases of Alzheimer's disease. Curr Top Dev Biol.

[2] Bradley-Whitman MA, Lovell MA (2015). Biomarkers of lipid peroxidation in Alzheimer disease (AD): an update. Arch Toxicol.

[3] Rojas-Gutierrez E, Munoz-Arenas G, Trevino S, Espinosa B, Chavez R, Rojas K (2017). Alzheimer's disease and metabolic syndrome: A link from oxidative stress and inflammation to neurodegeneration. Synapse.

[4] Shieh JC, Huang PT, Lin YF (2020). Alzheimer's Disease and Diabetes: Insulin Signaling as the Bridge Linking Two Pathologies. Mol Neurobiol.

[5] Fan YC, Chou CC, You SL, Sun CA, Chen CJ, Bai CH**.** Impact of Worsened Metabolic Syndrome on the Risk of Dementia: A Nationwide Cohort Study. J Am Heart Assoc. 2017;6(9):e004749. 10.1161/JAHA.116.004749 PMC5634246 28899896

[6] Kim YJ, Kim SM, Jeong DH, Lee SK, Ahn ME, Ryu OH (2021). Associations between metabolic syndrome and type of dementia: analysis based on the National Health Insurance Service database of Gangwon province in South Korea. Diabetol Metab Syndr.

[7] Lovell MA, Xie C, Markesbery WR (2001). Acrolein is increased in Alzheimer's disease brain and is toxic to primary hippocampal cultures. Neurobiol Aging.

[8] Alfarhan M, Jafari E, Narayanan SP**.** Acrolein: A Potential Mediator of Oxidative Damage in Diabetic Retinopathy. Biomolecules. 2020;10(11):1579. 10.3390/biom10111579 PMC7699716 33233661

[9] Chen C, Lu J, Peng W, Mak MS, Yang Y, Zhu Z (2022). Acrolein, an endogenous aldehyde induces Alzheimer's disease-like pathologies in mice: A new sporadic AD animal model. Pharmacol Res.

[10] Chen C, Chen Y, Lu J, Chen Z, Wang C, Pi R (2021). Acrolein-conjugated proteomics in brains of adult C57BL/6 mice chronically exposed to acrolein and aged APP/PS1 transgenic AD mice. Toxicol Lett.

[11] Moghe A, Ghare S, Lamoreau B, Mohammad M, Barve S, McClain C (2015). Molecular mechanisms of acrolein toxicity: relevance to human disease. Toxicol Sci.

[12] Kwon D (2022). Guardians of the brain: how a special immune system protects our grey matter. Nature.

[13] Angiolillo A, Gandaglia A, Arcaro A, Carpi A, Gentile F, Naso F (2021). Altered Blood Levels of Anti-Gal Antibodies in Alzheimer's Disease: A New Clue to Pathogenesis?. Life (Basel).

[14] Wang BZ, Zailan FZ, Wong BYX, Ng KP, Kandiah N (2020). Identification of novel candidate autoantibodies in Alzheimer's disease. Eur J Neurol.

[15] DeMarshall CA, Nagele EP, Sarkar A, Acharya NK, Godsey G, Goldwaser EL (2016). Detection of Alzheimer's disease at mild cognitive impairment and disease progression using autoantibodies as blood-based biomarkers. Alzheimers Dement (Amst).

[16] Agrawal S, Abud EM, Snigdha S, Agrawal A (2018). IgM response against amyloid-beta in aging: a potential peripheral protective mechanism. Alzheimers Res Ther.

[17] Liu YH, Wang J, Li QX, Fowler CJ, Zeng F, Deng J, et al. Association of naturally occurring antibodies to ß-amyloid with cognitive decline and cerebral amyloidosis in Alzheimer's disease. Sci Adv. 2021;7(1):eabb0457. 10.1126/sciadv.abb0457 PMC7775771 33523832

[18] Renuka Sanotra M, Huang WC, Silver S, Lin CY, Chang TC, Nguyen DPQ (2022). Serum levels of 4-hydroxynonenal adducts and responding autoantibodies correlate with the pathogenesis from hyperglycemia to Alzheimer's disease. Clin Biochem.

[19] Lin CY, Sheu JJ, Tsai IS, Wang ST, Yang LY, Hsu IU (2018). Elevated IgM against Nepsilon-(Carboxyethyl)lysine-modified Apolipoprotein A1 peptide 141–147 in Taiwanese with Alzheimer's disease. Clin Biochem.

[20] Tsai YF, Yang DJ, Ngo TH, Shih CH, Wu YF, Lee CK (2019). Ganglioside Hp-s1 Analogue Inhibits Amyloidogenic Toxicity in Alzheimer's Disease Model Cells. ACS Chem Neurosci.

[21] Ting LL, Lu HT, Yen SF, Ngo TH, Tu FY, Tsai IS (2019). Expression of AHI1 Rescues Amyloidogenic Pathology in Alzheimer's Disease Model Cells. Mol Neurobiol.

[22] Guze SB**.** Diagnostic and Statistical Manual of Mental Disorders, 4th ed. (DSM-IV). Am J Psychiatry. 1995;152(8):1228.

[23] McKhann G, Drachman D, Folstein M, Katzman R, Price D, Stadlan EM (1984). Clinical diagnosis of Alzheimer's disease: report of the NINCDS-ADRDA Work Group under the auspices of Department of Health and Human Services Task Force on Alzheimer's Disease. Neurology.

[24] Button EB, Robert J, Caffrey TM, Fan J, Zhao W, Wellington CL (2019). HDL from an Alzheimer's disease perspective. Curr Opin Lipidol.

[25] Chen GF, Xu TH, Yan Y, Zhou YR, Jiang Y, Melcher K (2017). Amyloid beta: structure, biology and structure-based therapeutic development. Acta Pharmacol Sin.

[26] Liu J, Yang B, Ke J, Li W, Suen WC (2016). Antibody-Based Drugs and Approaches Against Amyloid-beta Species for Alzheimer's Disease Immunotherapy. Drugs Aging.

[27] Uen YH, Liao CC, Lin JC, Pan YH, Liu YC, Chen YC (2015). Analysis of differentially expressed novel post-translational modifications of plasma apolipoprotein E in Taiwanese females with breast cancer. J Proteomics.

[28] Sheu JJ, Yang LY, Sanotra MR, Wang ST, Lu HT, Kam RSY (2020). Reduction of AHI1 in the serum of Taiwanese with probable Alzheimer's disease. Clin Biochem.

[29] Lasse M, Stampfli AR, Orban T, Bothara RK, Gerrard JA, Fairbanks AJ (2021). Reaction dynamics and residue identification of haemoglobin modification by acrolein, a lipid-peroxidation by-product. Biochim Biophys Acta Gen Subj.

[30] Zhang X, Tong T, Chang A, Ang TFA, Tao Q, Auerbach S, et al. Midlife lipid and glucose levels are associated with Alzheimer's disease. Alzheimers Dement. 2023;19(1):181–93. 10.1002/alz.12641 PMC10078665 35319157

[31] Dimache AM, Salaru DL, Sascau R, Statescu C (2021). The Role of High Triglycerides Level in Predicting Cognitive Impairment: A Review of Current Evidence. Nutrients.

[32] Cutuli D, De Bartolo P, Caporali P, Laricchiuta D, Foti F, Ronci M (2014). n-3 polyunsaturated fatty acids supplementation enhances hippocampal functionality in aged mice. Front Aging Neurosci.

[33] Mett J (2021). The Impact of Medium Chain and Polyunsaturated omega-3-Fatty Acids on Amyloid-beta Deposition, Oxidative Stress and Metabolic Dysfunction Associated with Alzheimer's Disease. Antioxidants (Basel).

[34] Bradley MA, Markesbery WR, Lovell MA (2010). Increased levels of 4-hydroxynonenal and acrolein in the brain in preclinical Alzheimer disease. Free Radic Biol Med.

[35] Endo R, Uchiyama K, Lim SY, Itakura M, Adachi T, Uchida K (2021). Recognition of acrolein-specific epitopes by B cell receptors triggers an innate immune response. J Biol Chem.

[36] Selkoe DJ (2021). Treatments for Alzheimer's disease emerge. Science.

[37] Lu Y, Liu J, Tong A, Lu Y, Lv L (2021). Interconversion and Acrolein-Trapping Capacity of Cardamonin/Alpinetin and Their Metabolites In Vitro and In Vivo. J Agric Food Chem.

[38] Toda N, Okamura T (2016). Cigarette smoking impairs nitric oxide-mediated cerebral blood flow increase: Implications for Alzheimer's disease. J Pharmacol Sci.

[39] Durazzo TC, Mattsson N, Weiner MW, Alzheimer's Disease Neuroimaging I**.** Smoking and increased Alzheimer's disease risk: a review of potential mechanisms. Alzheimers Dement. 2014;10(3 Suppl):S122–45. 10.1016/j.jalz.2014.04.009 PMC4098701 24924665

