# Supplementary material for: Acrolein adducts and responding autoantibodies correlate with metabolic disturbance in Alzheimer’s disease
Source: Alzheimers Res Ther. 2023 Jun 22;15:115. doi: 10.1186/s13195-023-01261-2 (PMC10286356; doi:10.1186/s13195-023-01261-2)
Supplement: Supplementary file 1 — Additional file 1: Table S1. Pearson correlation of age with the tested variables. Table S2. Statistical significance of demographic and clinical data (p value) between the groups. Table S3. Correlation of all tested variables. Fig. S1. Acrolein structure. PubChem, Acrolein. National Center for Biotechnology Information, 2022. Retrieved from https://pubchem.ncbi.nlm.nih.gov/compound/Acrolein. Fig. S2. Levels of IgG autoantibodies against native Aß peptides in human serum. Fig. S3. Levels of IgM autoantibodies against native Aß peptides in human serum. [file 13195_2023_1261_MOESM1_ESM.pdf]

Table S1. Pearson correlation of age with the tested variables

Variable	Ctrl (n = 142)		AD (n = 117)	
	Correlation Coefficient (r)	*p* value	Correlation Coefficient (r)	*p* value
ALT (IU/L)	-0.151	NS	0.0451	NS
Creatinine (mg/dL)	-0.0259	NS	0.0646	NS
Fasting Glucose (mg/dL)	-0.121	NS	0.00676	NS
HbA1c (%)	-0.234	NS	0.0732	NS
HDL-C (mg/dL)	**-0.244**	**0.0289**	0.0275	NS
LDL-C (mg/dL)	-0.181	NS	-0.214	NS
Total Cholesterol (mg/dL)	**-0.198**	**0.0459**	-0.0949	NS
Total protein (g/L)	-0.0325	NS	-0.0981	NS
Triglycerides (mg/dL)	0.0805	NS	0.153	NS
Aβ	0.13	NS	0.0786	NS
Acrolein adducts	-0.0886	NS	0.147	NS

The *p* values represent the probability of being wrong in the association between the variables and shown as NS with no statistical significance ($p \geq 0.05$). Aβ, amyloid-beta; AD, Alzheimer's disease; ALT, alanine transaminase; Ctrl, control; HbA1c, glycohemoglobin A1; HDL-C, high-density lipoprotein-cholesterol; LDL-C, low-density lipoprotein-cholesterol; NS, no statistical significance.

Table S2. Statistical significance of demographic and clinical data (*p* value) between the groups.

Testing group	MetS			AD-N		AD- M
Pairing group	HC	AD-N	AD- M	HC	AD- M	HC
Gender	NS	NS	NS	NS	NS	NS
Age	NS	<0.001	**<0.001**	**<0.001**	No	<0.001
ALT	NS	NS	NS	NS	NS	NS
Creatinine	NS	NS	**0.016**	NS	NS	0.009
Fasting glucose	**<0.001**	0.008	NS	NS	**<0.001**	<0.001
HbA1c	NS	0.004	NS	NS	**0.007**	NS
HDL-C	NS	0.039	**0.003**	NS	**<0.001**	<0.001
LDL-C	NS	NS	**0.006**	NS	NS	NS
Total cholesterol	NS	NS	NS	NS	NS	NS
Total protein	NS	NS	NS	NS	NS	NS
Triglycerides	**0.001**	<0.001	NS	NS	**<0.001**	<0.001

The *p* values with no statistical significance ($p \geq 0.05$) were shown as NS. AD-M, Alzheimer's disease with metabolic disturbance; AD-N, Alzheimer's disease with normal metabolism; ALT, alanine transaminase; HC, healthy control; HbA1c, glycohemoglobin A1; HDL-C, high-density lipoprotein-cholesterol; LDL-C, low-density lipoprotein-cholesterol; MetS, metabolic syndrome; NS, no statistical significance

Table S3. Correlation of all tested variables

	Creatinine	Glucose	HbA1c	HDL-C	LDL-C	T-Chol.	TP	TG	Aβ	**ACR**	NA1 IgG	NA2 IgG	NA1 IgM	NA2 IgM	ACRA1 IgG	ACRA2 IgG	ACRA1 IgM	ACRA2 IgM
ALT	-0.0725<br>0.32	0.00597<br>0.935	0.00153<br>0.989	-0.15<br>0.0854	0.0767<br>0.34	0.0322<br>0.669	**0.152<br>0.0325**	-0.0244<br>0.733	0.0671<br>0.348	0.0686<br>0.337	0.0667<br>0.351	0.0269<br>0.707	0.0621<br>0.385	-0.00101<br>0.989	-0.0433<br>0.545	-0.0548<br>0.443	0.00342<br>0.962	-0.0449<br>0.53
Creatinine		**0.194<br>0.00558**	-0.0579<br>0.573	**-0.219<br>0.0121**	**-0.159<br>0.0452**	-0.0481<br>0.524	-0.00312<br>0.964	**0.164<br>0.0176**	0.0706<br>0.308	**0.188<br>0.00627**	0.0558<br>0.421	-0.0352<br>0.612	**-0.146<br>0.0349**	-0.102<br>0.141	-0.0288<br>0.678	-0.0735<br>0.289	-0.0702<br>0.311	0.0137<br>0.843
Glucose			**0.392<br>8.73E-05**	**-0.219<br>0.011**	-0.112<br>0.161	-0.00177<br>0.981	0.00599<br>0.932	**0.24<br>0.00049**	0.0591<br>0.396	**0.192<br>0.0054**	-0.00527<br>0.94	-0.0103<br>0.883	-0.0354<br>0.612	-0.0953<br>0.171	0.0216<br>0.756	0.0745<br>0.285	-0.0473<br>0.498	0.0458<br>0.511
HbA1c				-0.0499<br>0.696	0.00232<br>0.983	-0.0264<br>0.809	-0.0453<br>0.656	**0.265<br>0.00794**	-0.121<br>0.234	-0.0241<br>0.813	0.0091<br>0.929	-0.0601<br>0.555	0.0761<br>0.454	-0.074<br>0.467	-0.00836<br>0.935	-0.0371<br>0.715	**0.301<br>0.00248**	0.136<br>0.18
HDL-C					**0.413<br>6.09E-06**	**0.375<br>1.03E-05**	0.117<br>0.178	**-0.341<br>5.23E-05**	**-0.234<br>0.00624**	**-0.354<br>2.6E-05**	-0.0923<br>0.287	-0.0285<br>0.743	-0.119<br>0.17	-0.0341<br>0.695	**-0.224<br>0.00906**	-0.0369<br>0.671	-0.127<br>0.142	-0.043<br>0.62
LDL-C						**0.729<br>2.81E-26**	0.0166<br>0.834	-0.0196<br>0.805	**-0.228<br>0.00355**	**-0.209<br>0.0075**	0.00386<br>0.961	0.0139<br>0.86	0.0183<br>0.817	0.0631<br>0.425	0.0279<br>0.724	0.0312<br>0.694	0.0552<br>0.486	-0.0334<br>0.673
T-Chol.							0.0305<br>0.681	**0.248<br>0.0007**	**-0.175<br>0.0173**	-0.133<br>0.0718	-0.0521<br>0.483	-0.0753<br>0.31	-0.00689<br>0.926	0.0285<br>0.701	0.0519<br>0.484	0.0276<br>0.71	0.0247<br>0.739	-0.0629<br>0.396
TP								-0.0152<br>0.808	-0.0452<br>0.469	**-0.25<br>4.8E-05**	**-0.163<br>0.00856**	0.0313<br>0.617	-0.117<br>0.0607	0.0759<br>0.224	**0.266<br>0.0000145**	0.0357<br>0.568	-0.0145<br>0.817	0.0822<br>0.188
TG									0.02<br>0.748	**0.156<br>0.0119**	0.0391<br>0.531	0.0888<br>0.154	0.0409<br>0.512	0.0599<br>0.337	0.0993<br>0.111	0.0121<br>0.847	-0.0565<br>0.365	0.0506<br>0.417
Aβ										**0.29<br>2E-06**	**0.251<br>0.0000435**	**0.162<br>0.00913**	0.0788<br>0.206	0.0297<br>0.634	**0.182<br>0.00336**	0.0267<br>0.669	0.0604<br>0.333	0.0176<br>0.779
**ACR**											**0.21<br>0.000672**	-0.00214<br>0.973	0.0215<br>0.73	-0.102<br>0.1	0.0184<br>0.768	-0.101<br>0.105	-0.00265<br>0.966	**-0.141<br>0.0232**
NA1 IgG												**0.198<br>0.00139**	**0.326<br>7.91E-08**	0.11<br>0.0777	**0.173<br>0.00525**	0.0866<br>0.165	-0.0623<br>0.318	**-0.124<br>0.0464**
NA2 IgG													**0.188<br>0.00239**	**0.34<br>1.95E-08**	**0.324<br>9.87E-08**	**0.135<br>0.0297**	0.0206<br>0.742	0.0712<br>0.254
NA1 IgM														**0.387<br>1.14E-10**	0.0531<br>0.395	0.0222<br>0.722	**0.285<br>0.00000304**	0.116<br>0.0616
NA2 IgM															**0.152<br>0.0145**	0.0196<br>0.753	**0.271<br>0.00000987**	0.0941<br>0.131
ACRA1 IgG																**0.289<br>0.00000216**	0.0959<br>0.124	-0.0366<br>0.558
ACRA2 IgG																	0.0151<br>0.808	**0.203<br>0.00101**
ACRA1 IgM																		**0.273<br>0.00000857**

In each cell, the correlation coefficient (r) was shown at the top and the *p* value at the bottom. The values with statistical significance ($p < 0.05$) were shown in **bold red**. Aβ, amyloid-beta; ACR, acrolein adducts; ACRA1, acrolein-modified amyloid-beta residues 1-16; ACRA2, acrolein-modified amyloid-beta residues 17-28; ALT, alanine transaminase; HbA1c, glycohemoglobin A1; HDL-C, high-density lipoprotein-cholesterol; LDL-C, low-density lipoprotein-cholesterol; NA1, native amyloid-beta residues 1-16; NA2, native amyloid-beta residues 17-28; T-Chol, total cholesterol; TG, triglycerides; TP, total protein.

C_3H_4O

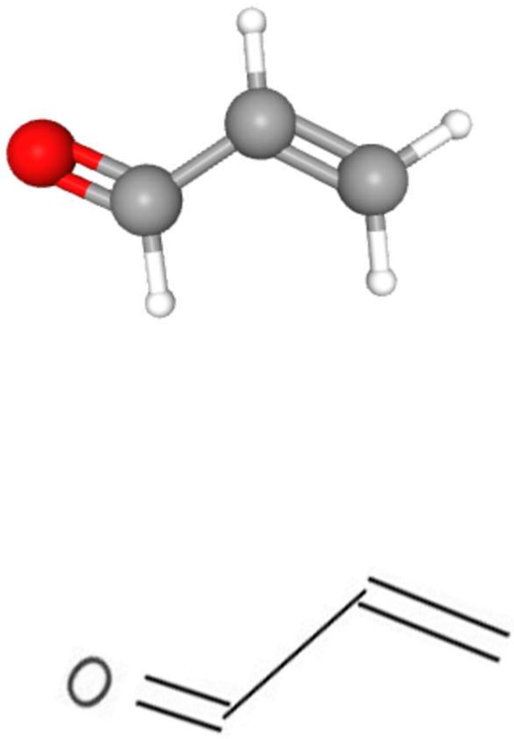

Fig. S1. Acrolein structure. PubChem, *Acrolein.* National Center for Biotechnology Information, 2022. **Retrieved from** **https://pubchem.ncbi.nlm.nih.gov/compound/Acrolein.**

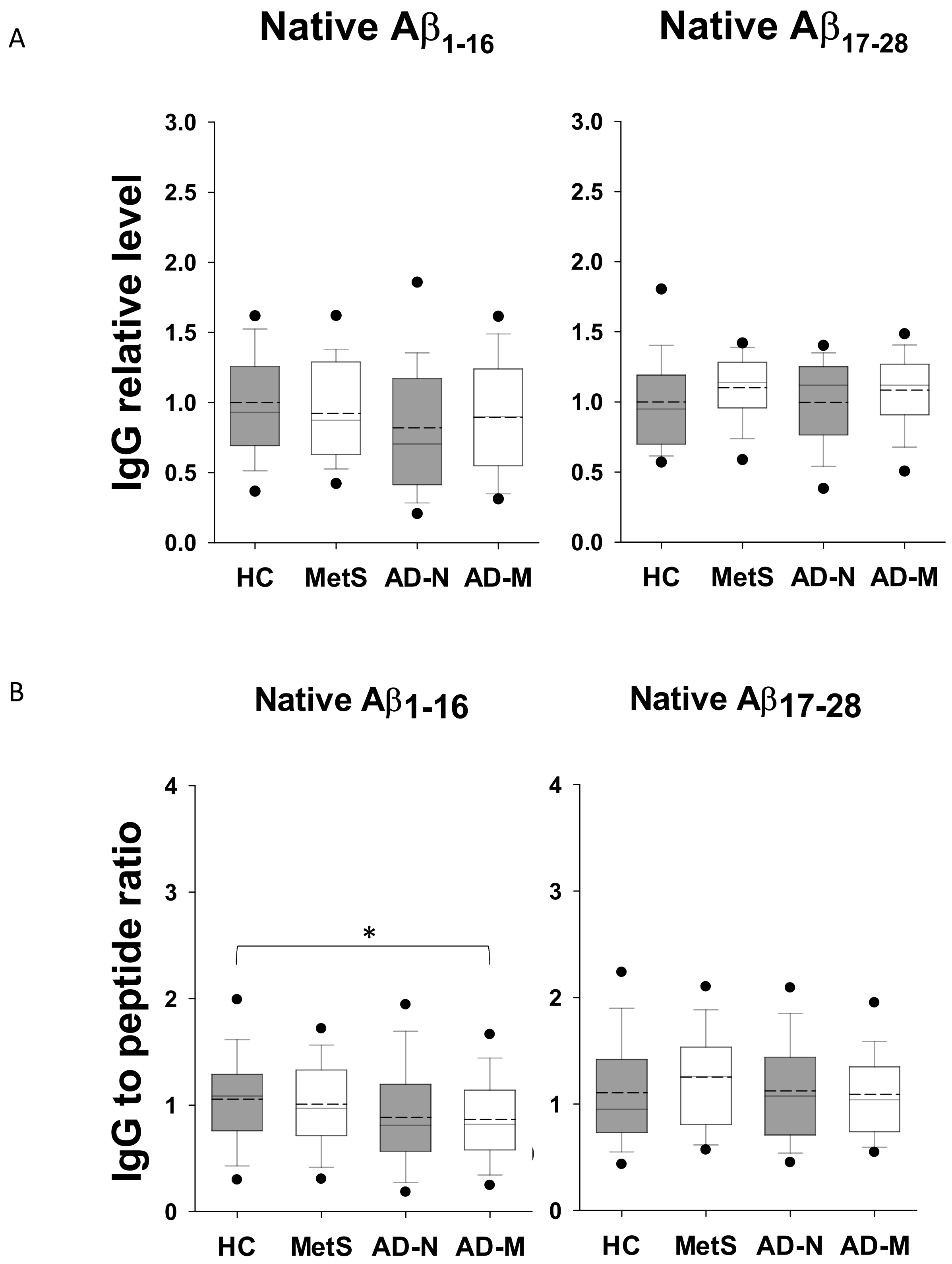

Fig. S2. **Levels of IgG autoantibodies against native Aβ peptides in human serum. A**. The relative levels of IgG autoantibody recognizing native $A\beta_{1\text{-}16}$ and $A\beta_{17\text{-}28}$ peptides in each group. All the values were normalized with the mean of healthy control (HC). **B**. The ratio of the responding IgG to acrolein adducts of each subject in each group. In the box plots, the dots represent 5th and 95th percentiles. The error bars cover the 10th to 90th percentiles and the box covers 25th to 75th percentiles. The solid and dash lines within the box represent the median and mean values, respectively. Aβ, amyloid beta; AD-M, Alzheimer's disease with metabolic disturbance; AD-N, Alzheimer's disease with normal metabolism; HC, healthy control; MetS, metabolic syndrome. * $p < 0.05$,

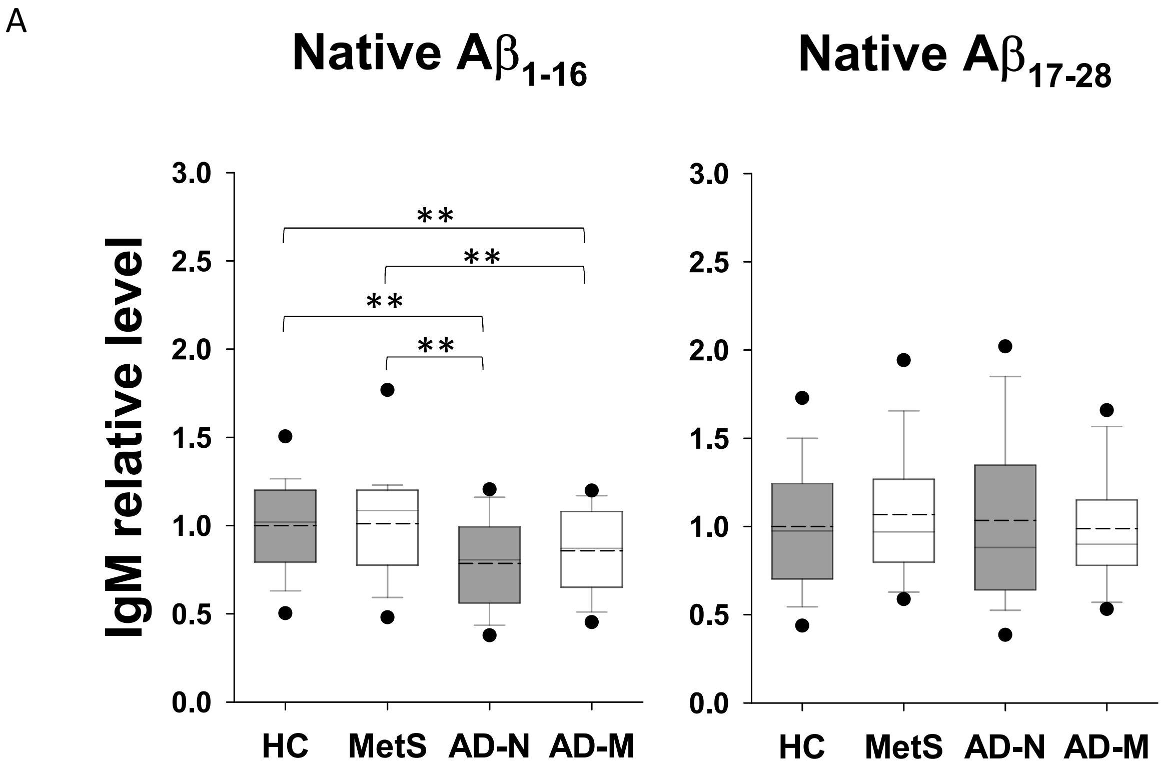

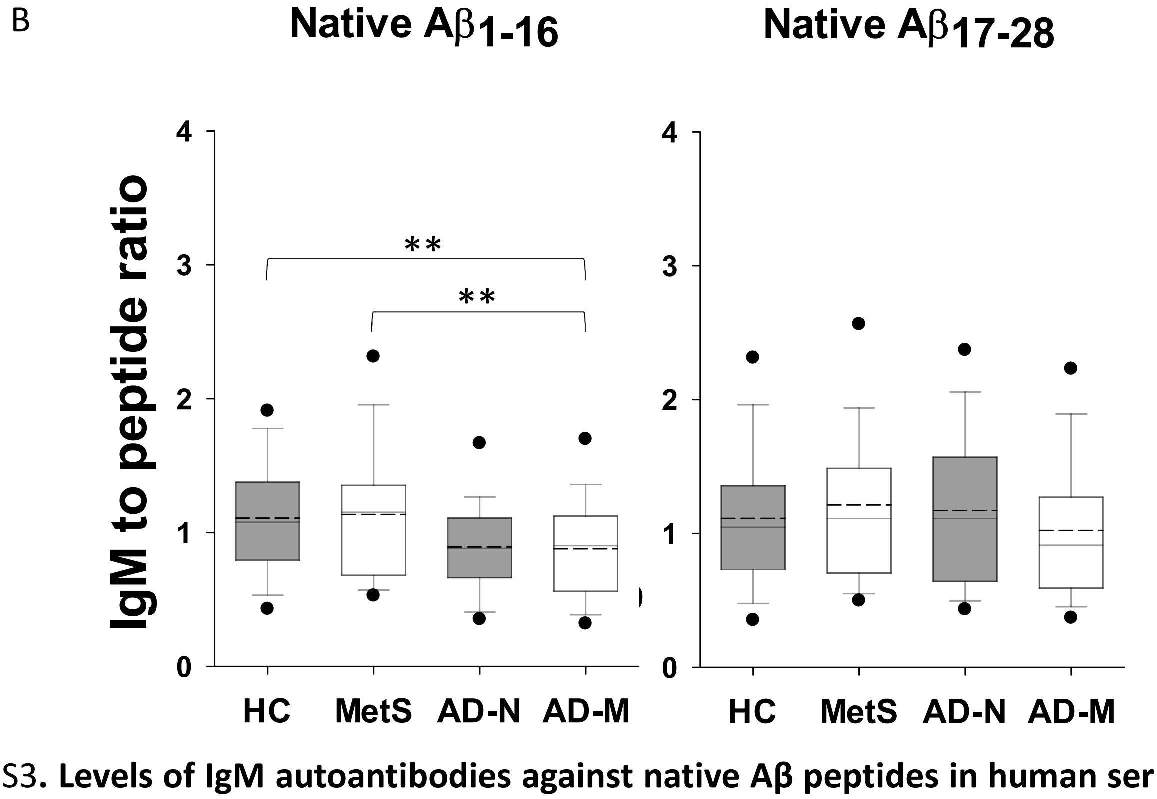

Fig. S3**. Levels of IgM autoantibodies against native Aβ peptides in human serum**. The relative levels of IgM autoantibody recognizing native $A\beta_{1\text{-}16}$ and $A\beta_{17\text{-}28}$ peptides in each group. All the values were normalized with the mean of healthy control (HC). **B**. The ratio of the responding IgM to acrolein adducts of each subject in each group. In the box plots, the dots represent 5th and 95th percentiles. The error bars cover the 10th to 90th percentiles and the box covers 25th to 75th percentiles. The solid and dash lines within the box represent the median and mean values, respectively. Aβ, amyloid beta; AD-M, Alzheimer's disease with metabolic disturbance; AD-N, Alzheimer's disease with normal metabolism; HC, healthy control; MetS, metabolic syndrome. ** $p < 0.01$
